# Temporal encoding in deep reinforcement learning agents

**DOI:** 10.1038/s41598-023-49847-y

**Published:** 2023-12-15

**Authors:** Dongyan Lin, Ann Zixiang Huang, Blake Aaron Richards

**Affiliations:** 1 Integrated Program in Neuroscience, McGill University, Montreal, QC H3A 0G4 Canada; 2 Mila, Montreal, QC H2S 3H1 Canada; 3 School of Computer Science, McGill University, Montreal, QC H3A 0G4 Canada; 4 Department of Neurology and Neurosurgery, Montreal Neurological Institute, McGill University, Montreal, QC H3A 0G4 Canada; 5 Learning in Machines and Brains Program, Canadian Institute for Advanced Research, Toronto, ON M5G 1M1 Canada

**Keywords:** Network models, Intelligence, Computer science

## Abstract

Neuroscientists have observed both cells in the brain that fire at specific points in time, known as “time cells”, and cells whose activity steadily increases or decreases over time, known as “ramping cells”. It is speculated that time and ramping cells support temporal computations in the brain and carry mnemonic information. However, due to the limitations in animal experiments, it is difficult to determine how these cells really contribute to behavior. Here, we show that time cells and ramping cells naturally emerge in the recurrent neural networks of deep reinforcement learning models performing simulated interval timing and working memory tasks, which have learned to estimate expected rewards in the future. We show that these cells do indeed carry information about time and items stored in working memory, but they contribute to behavior in large part by providing a dynamic representation on which policy can be computed. Moreover, the information that they do carry depends on both the task demands and the variables provided to the models. Our results suggest that time cells and ramping cells could contribute to temporal and mnemonic calculations, but the way in which they do so may be complex and unintuitive to human observers.

## Introduction

The neural computations underlying core cognitive functions such as navigation, memory, and timing, have long been a central question in neuroscience. Many of these cognitive functions have been linked to the tuning of neurons’ firing rates in the medial temporal lobes. For example, place cells in the hippocampus are considered a neural substrate for navigation^1^, and due to their special properties such as contextual remapping^2^ and offline replay^3^, they have also been considered important to episodic memory. As the temporal analogue of place cells, several recent studies have identified neurons in hippocampus CA1 and CA3 that tile the interval between discontiguous events by firing sequentially at successive moments in time, suggesting that these “time cells” support the organization of memory by encoding time^4–10^. The subsequent observations of such time cells throughout the brain in multiple mammalian species confirmed that this coding regime was wide-spread^6,10–29^. It can also be seen as complementary to the previously reported ramping-based model for tracking time, in which neurons estimate elapsed time using increasing or decreasing neuronal firing rates^30–35^. Interestingly, multiple studies have demonstrated that the same population of hippocampal time cells form distinct sequences during the mnemonic delay following the presentation of different sensory stimuli, suggesting a potential mechanism by which the hippocampus integrates information about “what” and “when” as part of the process of encoding memories^5,21,36^.

However, discrepancies in the current literature make it unclear whether time cells and ramping cells are causally responsible for temporally-organized behavior and working memory, or if they are an emergent phenomenon related to internal recurrent dynamics in circuits. For example, Salz et al.^9^ showed that these time cell sequences emerged from not only the mnemonic delayed alternative task but also a “looping task” that contained no memory load. Sabariego et al.^8^ reported that sequences formed by the hippocampal time cells during a spatial working memory task did not distinguish between different trial stimulus conditions. Toso et al.^29^ demonstrated a dissociation between time coding by ramping cells and time perception in the dorsolateral striatum of rats tasked with comparing the duration of two sequential vibrations. These findings hint at the possibility that time cells or ramping cells may not be how animals “compute time” for driving behavior, but could instead be related to more general computations that involve temporal information in recurrent calculations, such as estimating future-discounted value. Determining this would require difficult manipulations of task demands and highly targeted lesion studies of time and ramping cells to determine their involvement in behavior.

Luckily, computational models are not limited in this way—it is easy to manipulate the tasks given to models and lesions can be performed on specific cells at specific times. As such, in the present study, we use in-silico models, namely deep reinforcement learning (DRL) agents^37^, trained on simulated timing and working memory tasks to investigate the question of how time and ramping cells may contribute to behavior. We show that time cells and ramping cells, as defined in the neuroscience literature, emerge in the recurrent neural networks trained with reinforcement, even when performing the task does not require keeping track of time. As long as the network is calculating temporally discounted value, these cells emerge. And, time can be decoded from the activity of time and ramping cells equally well in networks trained to calculate time, networks trained on working memory tasks, and networks trained on simple stimulus-association tasks. Moreover, time cells and ramping cells carry other pieces of critical information for the task, such as stimulus identity and space. When we performed targeted lesion experiments to determine the role of time cells and ramping cells in the performance of these networks we found that time cells and ramping cells largely contribute to task performance courtesy of their contribution to recurrent dynamics, regardless of the task being performed. Moreover, when the networks operated in a spatial environment time and stimulus information were not encoded independently from space. Our data suggests that time cells and ramping cells could play a more subtle and complicated role in core cognitive functions, such as helping to estimate temporally discounted values via recurrent dynamics. Our results point to the need for more careful examination of the involvement of apparent, intuitive-to-human neural tuning curves, such as the tuning of firing rate to time elapsed, when trying to judge the role of different neurons in cognitive functions.

## Results

### Time cells and ramping cells naturally emerge in recurrent circuits trained on timing and working memory tasks

**Figure 1:**
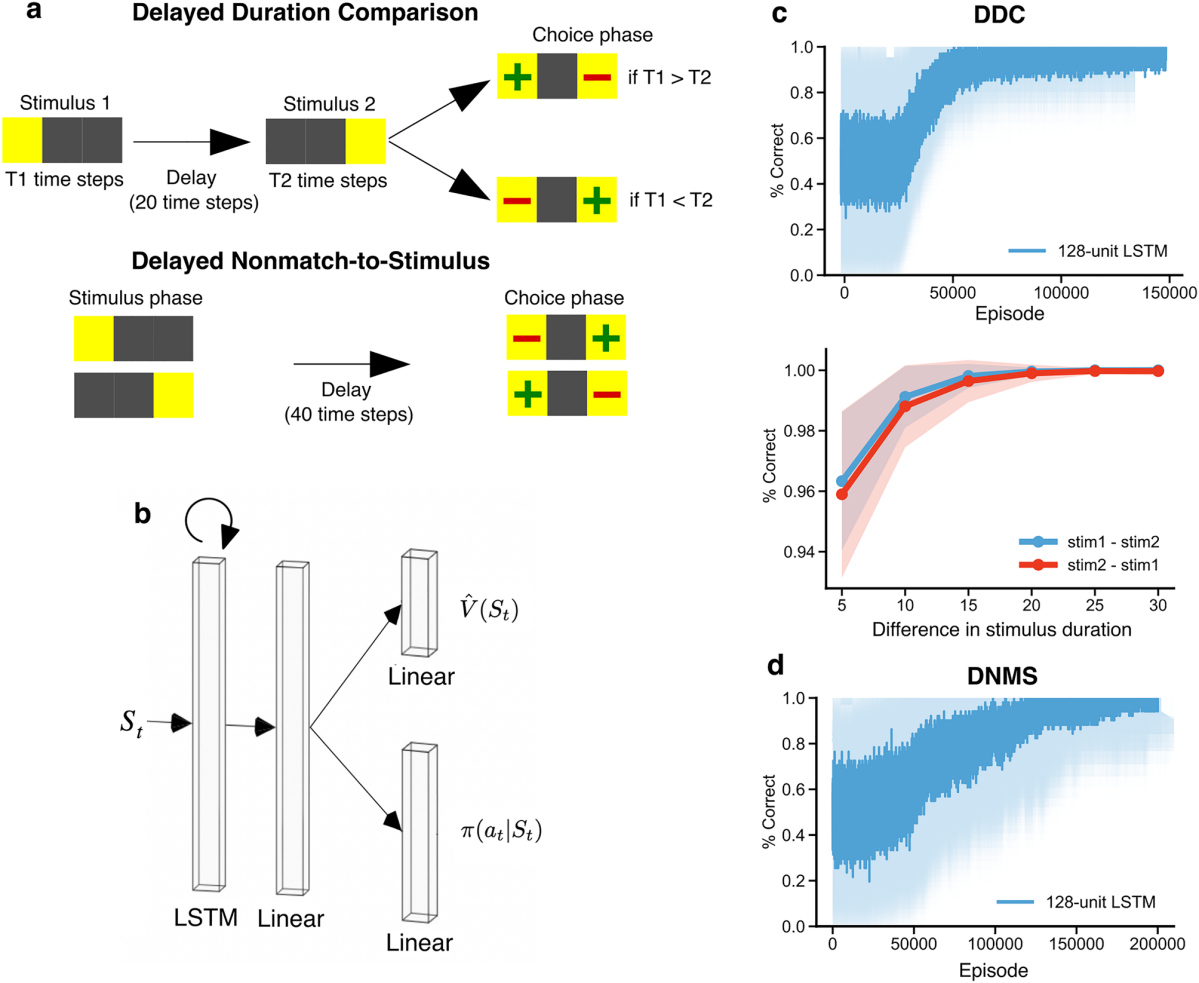
Training deep reinforcement learning (DRL) agents on simulated timing and working memory tasks. (**a**) Task schema. Top: Delayed duration comparison (DDC) task wherein the agent must choose the stimulus duration, T1 or T2, that is longer. Bottom: Delayed nonmatch-to-stimulus (DNMS) task wherein, after a delay in each trial, the agent must choose the option that is a non-match to the stimulus presented to receive a reward. (**b**) Agent architecture. At each time step, the agent received the input of the state of the environment ${S}_{t}$, and output the estimated state value $\widehat{V}({S}_{t})$ and the policy $\pi ({a}_{t}|{S}_{t})$. (**c**) Top: Performance of 50 agents with different random seeds on the DDC task over the course of training, measured by percentage of correct duration comparison. Bottom: Performance of 50 agents, after training on the DDC task, as a function of the difference between T1 and T2. (**d**) Performance of 50 agents on DNMS tasks over the course of training, measured by the percentage of nonmatch choice to the stimulus. Solid line and shaded area in (**c**) and (**d**) represent the average and standard deviation over 50 seeds, respectively.

In order to examine networks in conditions where time must be computed, we first simulated a Delayed Duration Comparison (DDC) task, an interval duration judgment task for which time cells and ramping cells have been observed in the hippocampus^22^ and striatum^29^. In each episode, the agent receives two stimuli of varying length presented sequentially, separated by a fixed-length delay period (Fig. 1a, top). The duration of stimulus 1 and stimulus 2 was sampled uniformly among 7 equally spaced stimulus lengths between 10 and 40 s (10, 15, 20, and so on), under the constraint that the two stimuli must have different durations. After the stimulus presentation, the agent makes a response to indicate which stimulus was longer and receives a reward for the correct response. Similar to animal training, we used a reinforcement learning paradigm. Here, we trained DRL agents that had an actor-critic architecture (Fig. 1b). The models assumed rate-based coding, i.e., each unit had a real-valued activity. In any aggregate data presented here we trained 50 agents with different random seeds for statistical analyses. At each time step, the agents received the stimulus as input (one-hot encoded), which was passed through a layer of recurrent units and a layer of linear units, before outputting a value estimate and a policy (Fig. 1b; see “Methods”). Over episodes the agents’ performance improved, reaching almost 100% correct eventually (Fig. 1c, top). The performance depended on the difference in the durations for stimulus 1 and stimulus 2 (the greater the difference the higher the performance), but it did not depend on which of the stimuli was longer (Fig. 1c, bottom), similar to behavioral results in animals^38^.

We also simulated the delayed non-match-to-stimulus (DNMS) task, an episodic working memory task that does not require keeping track of time, but in which time cells have been observed in the hippocampus of location-fixed, trained animals during the delay period^36^. In each episode, the DRL agents received one of two possible stimuli, followed by a stimulus-free delay, after which the agents had to choose whichever stimulus was not presented before the delay (i.e., non-match) to receive a reward (Fig. 1a, bottom). This task is fundamentally similar to other working memory tasks used in time cell studies that require the animal to hold a piece of information for a short period of time and make a decision based on the information, such as delayed match-to-sample tasks^36,39^, object-pairing tasks^5,40^ and delayed alternation tasks^4,6,8,9,26,41,42^. We used the same agent architecture as for the DDC task (Fig. 1b). The agents were also able to learn this task well, getting close to 100% accuracy by the end of training (Fig. 1d).

**Figure 2:**
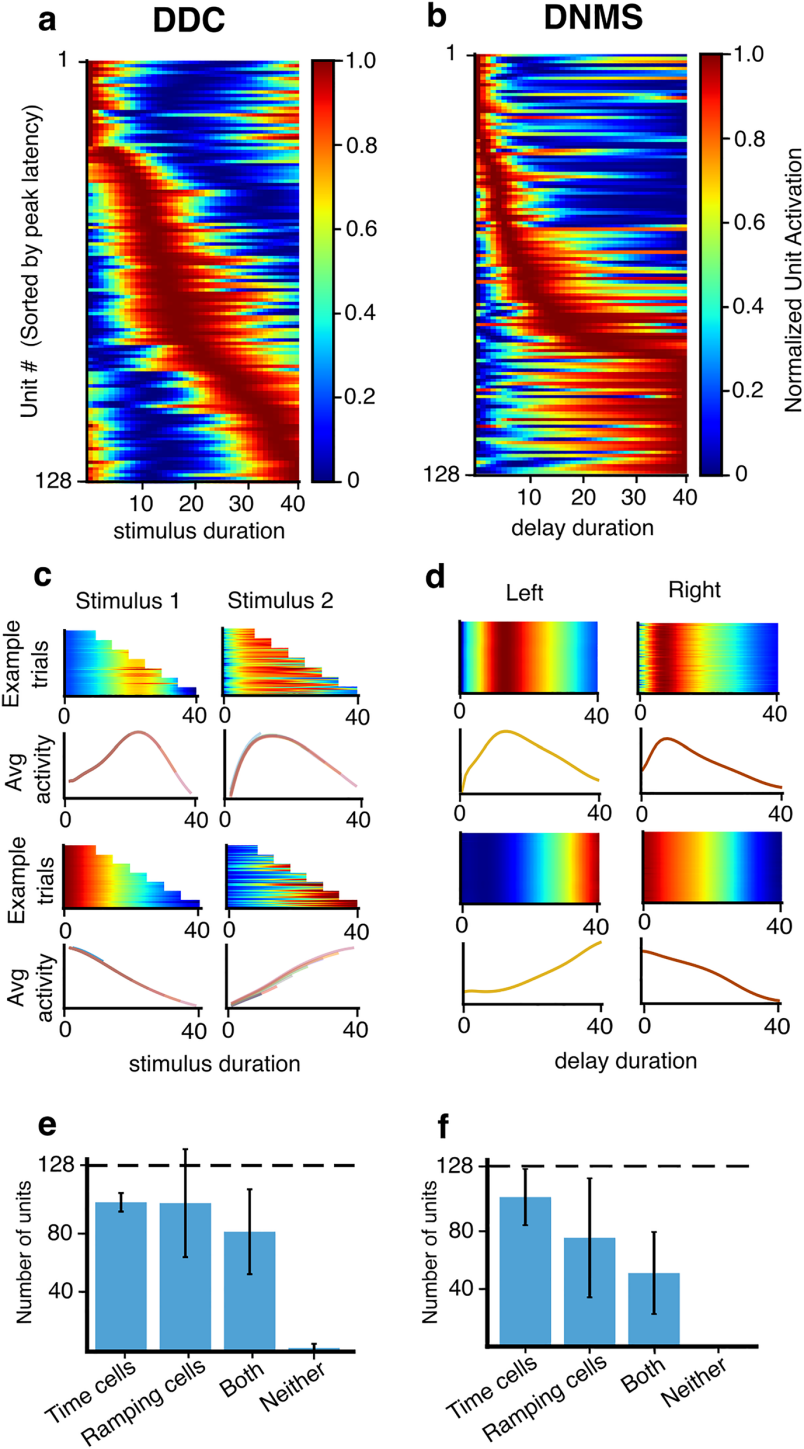
Ramping cells and time cells emerge in the recurrent units in DRL agents trained on cognitive tasks. (**a**) In an example agent trained on the DDC task, the heatmap shows the activity of the RNN units of the agent during the stimulus presentation, averaged across both stimuli for all durations. Each row shows the trial-averaged activity of a single unit normalized to its minimum (blue) and maximum (red) activity throughout the recorded episodes. Rows in each panel are sorted by the latency to the peak trial-averaged activity of units. (**b**) Same as (**a**), but for the RNN activity during the delay period in an example agent trained on the DNMS task. (**c**) An example time cell (top row) and an example ramping cell (bottom row) during the presentation of stimulus 1 (left column) and stimulus 2 (right column) in the DDC task. In each panel, the heatmap shows normalized RNN activity during the stimulus presentation in 100 consecutive example trials for each duration; the curves indicate the average activity across trials of the same duration throughout the recorded episodes, color-coded to indicate different durations. (**d**) Similar to (**c**), the heatmaps show the RNN activity during the delay period of the DNMS task in 100 example trials for each stimulus, the curves show the trial-averaged responses throughout the recorded episodes. (**e**) The number of units qualified as time cell, ramping cell, both time cell and ramping cell, and neither time cell nor ramping cell in each agent averaged across 50 agents trained on the DDC task. Error bars indicate the standard deviation. Dashed line indicates the total number of RNN units in the network (i.e., 128). (**f**) Same as (**e**) but for the DNMS task.

After the agents reached the performance of > 90% correct responses in both tasks we recorded the activity of the recurrent units in the neural network for 5000 episodes with the weights frozen (i.e., no learning occurring), as an in-silico analogue of recording the firing rate of the population in a neural circuit. As in neural recordings, we observed the presence of activity that tiled the delay period in both the DDC and DNMS tasks (Fig. 2a,b), resembling the time cells and ramping cells observed in animals. Interestingly, the cell ensembles were characterized by a decrease in their temporal resolution over the delay, as reflected by the overrepresentation of the beginning of the delay period as well as an increase in the width of the temporal receptive field towards the end of the delay period (Fig. 2a,b), which is a phenomenon commonly observed in biological time cells across brain regions and species^5,6,9,12,15,17,18,39^. We then analyzed the recorded activity from the recurrent units with analyses commonly employed in time cell and ramping cell studies (see “Methods”). Briefly, time cells were defined as recurrent units whose trial-averaged temporal tuning curve had a significant temporal information^46^ compared to a shuffled distribution (Fig. 2c,d, top). Ramping cells were defined as recurrent units whose trial-averaged temporal tuning curve ramps either up or down during the interval of interest, as quantified by their fit to linear regression (Fig. 2c,d, bottom). In addition, to qualify as a ramping cell or time cell, a unit had to exhibit reliable activity patterns across trials to ensure that the trial-averaged temporal tuning curve was meaningful. Cells could qualify as both time and ramping cells if they met both criteria.

Across seeds, we observed the presence of both time cells and ramping cells for both tasks, as has been observed in animals and humans^15,18,23,31^. However, we observed that, while the number of units that qualified as time cells was similar for the DDC and the DNMS tasks, in the DDC task most seeds had a large number of ramping cells and thus a larger number of cells that qualified as both time cells and ramping cells (Fig. 2e,f). This is consistent with the traditional neuroscientific view that ramping activity in the cortical and striatal regions is involved in interval timing and temporal control of action^43–45^. Interestingly, in untrained networks we saw almost all units qualified as time cells, due to the fact that the change over time in the temporal tuning curve had significant temporal information, but no ramping cells and no difference between the two tasks (Fig. S1), suggesting that the presence of ramping cells and the difference between the two tasks in the trained networks was a result of the solutions discovered by learning. In line with this, we also observed that whereas transfer learning from the DDC task to the DNMS task was easy for the networks, transferring from the DNMS task to the DDC task was highly dependent on the random seeds used at initialization (Fig. S2). This implies that training on these two different tasks leads to different solutions, even though both reliably produce time and ramping cells. Altogether, these results show that even if performing the task does not require time tracking (DNMS), time and ramping cells can be present, but when time tracking is required by the task (DDC), more ramping cells are present. And, when we trained networks without resetting the hidden unit activity in between trials we observed even fewer ramping cells in the DNMS task (Fig. S3). This suggests that ramping cells can be useful for timing calculations, but can be present even when performing the task does not require time tracking. As well, time cells (as defined by the criteria used in neuroscience studies) appear to be a natural phenomena in recurrent networks, even untrained networks (Fig. S1), which suggests that their presence is not necessarily indicative of any temporal calculations.

### Time and ramping cells emerge based on value calculation demands

**Figure 3:**
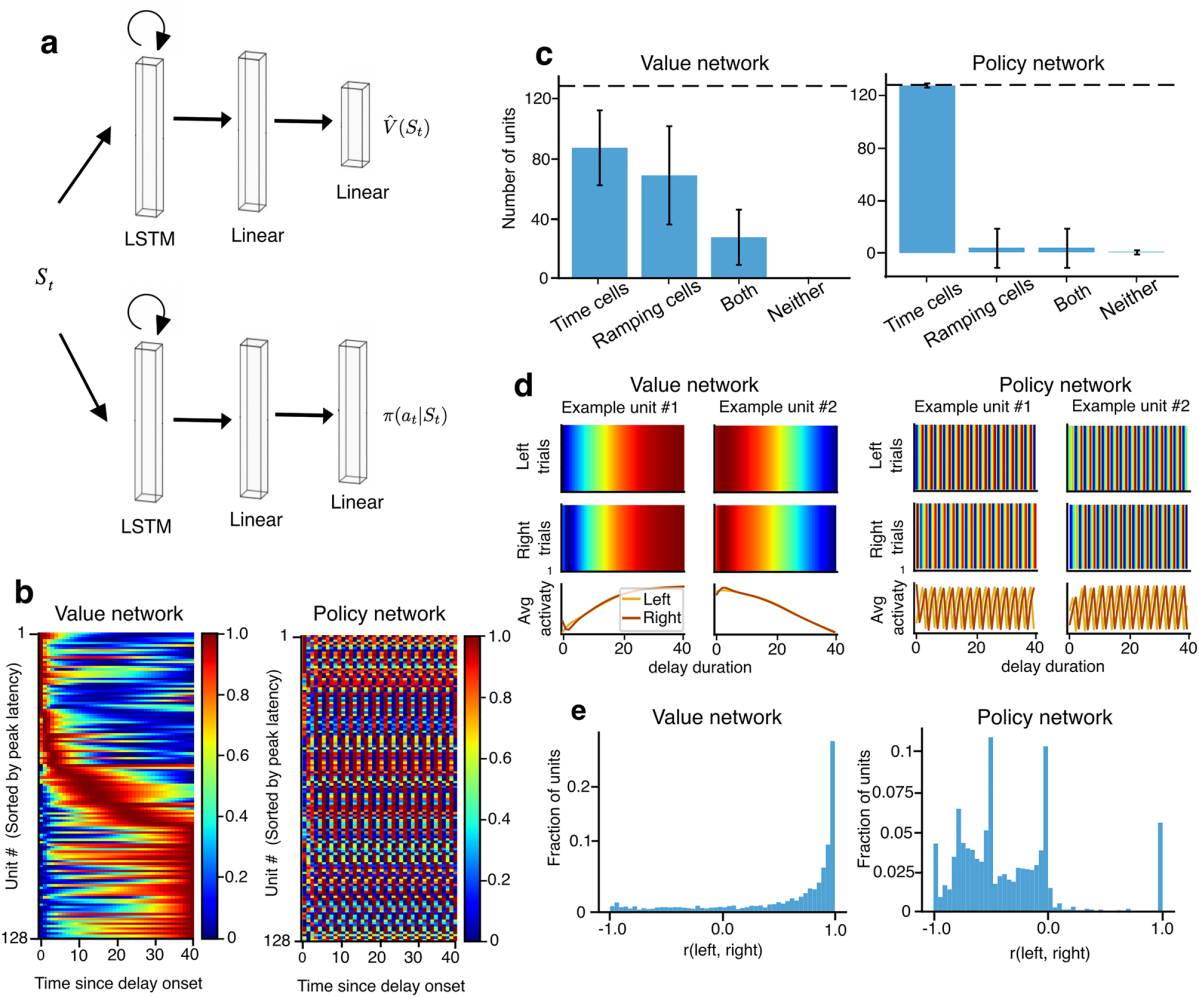
Time and ramping cells only emerge when there is value calculation demand. (**a**) Agent architecture for separate actor (policy) and critic (value) pathways. The two pathways receive the same input, and calculate policy and value independently, without sharing representation. (**b**) Heatmaps show the trial-averaged RNN neural activity during the delay period in the value (left) and policy (right) networks in an example agent that successfully learned the DNMS task. (**c**) The number of units qualified as time cell, ramping cell, both time cell and ramping cell, and neither time cell nor ramping cell in the value network (left) and policy network (right) in each agent, averaged across all agents that successfully learned the DNMS task. Error bars indicate the standard deviation. Dashed line indicates the total number of RNN units in the network (i.e., 128). (**d**) Four example RNN units—two from the value network (left), two from the policy network (right)—in an agent that successfully learned the DNMS task. For each unit, the heatmaps show normalized RNN activity during the delay period in 100 consecutive trials for left (top panels) and right sample (middle panels). The bottom panels show the trial-averaged responses during the delay period in all recorded trials, for left trials (yellow curve) and right trials (brown curve). (**e**) Distributions of Pearson correlations between each RNN unit’s temporal tuning curves for left trials versus right trials in the value pathway (left) and policy pathway (right) of all agents that successfully learned the DNMS task.

One important factor to recognize, though, is that while performing the DNMS task does not require the agent to keep track of time, training the network on the task still requires the network to learn to estimate a temporally discounted value function. One possibility, then, is that this value learning is what drove the emergence of time cells and ramping cells in the DNMS task. To test this, we trained networks with separate pathways for the policy and value calculations (Fig. 3a). Many initializations of these networks struggled to learn the task, likely because the policy network benefits from the representations of state encouraged by the value function. Nonetheless, some seeds converged for this separate pathway architecture (Fig. S4). Interestingly, when we examined the activity of the recurrent units in the networks that did learn, the activity profiles of units in the two different pathways were radically different (Fig. 3b). In the value pathway, time cells and ramping cells emerged robustly again (Fig. 3c, left). In contrast, in the policy pathway, the units exhibited oscillatory activity during the delay, and almost no ramping cells emerged (Fig. 3c, right). As well, the cells that qualified as time cells according to the criteria in the policy pathway had a very different temporal receptive field than the time cells observed in the previous models with a single pathway.

We then examined the extent to which the units that qualified as time or ramping cells carried stimulus information. In the value pathway, the units no longer appeared to carry any information about whether the stimulus had appeared on the left or the right (Fig. 3d, left). In contrast the oscillatory units in the policy pathway did seem to distinguish between left and right (Fig. 3d, right). We quantified this using the Pearson correlation between the trial average activities for left trials versus right trials. As expected, given the qualitative appearance of the activity profiles of the units, the correlation between the left and right trials was very close to 1 for almost all units in the value pathway, and negative for most units in the policy pathway (Fig. 3e). These findings show that the emergence of single peaked time cells and ramping cells in the RNNs is driven by the temporal calculation involved in learning value estimation. They also show that the extent to which time and ramping cells carry stimulus information during a delay depends on there being a requirement for the RNN to remember a stimulus. A similar teleological origin for the behavior of time and ramping cells could therefore exist in the brain.

### Time cells and ramping cells contribute to timing calculation through value-based dynamic representation

**Figure 4:**
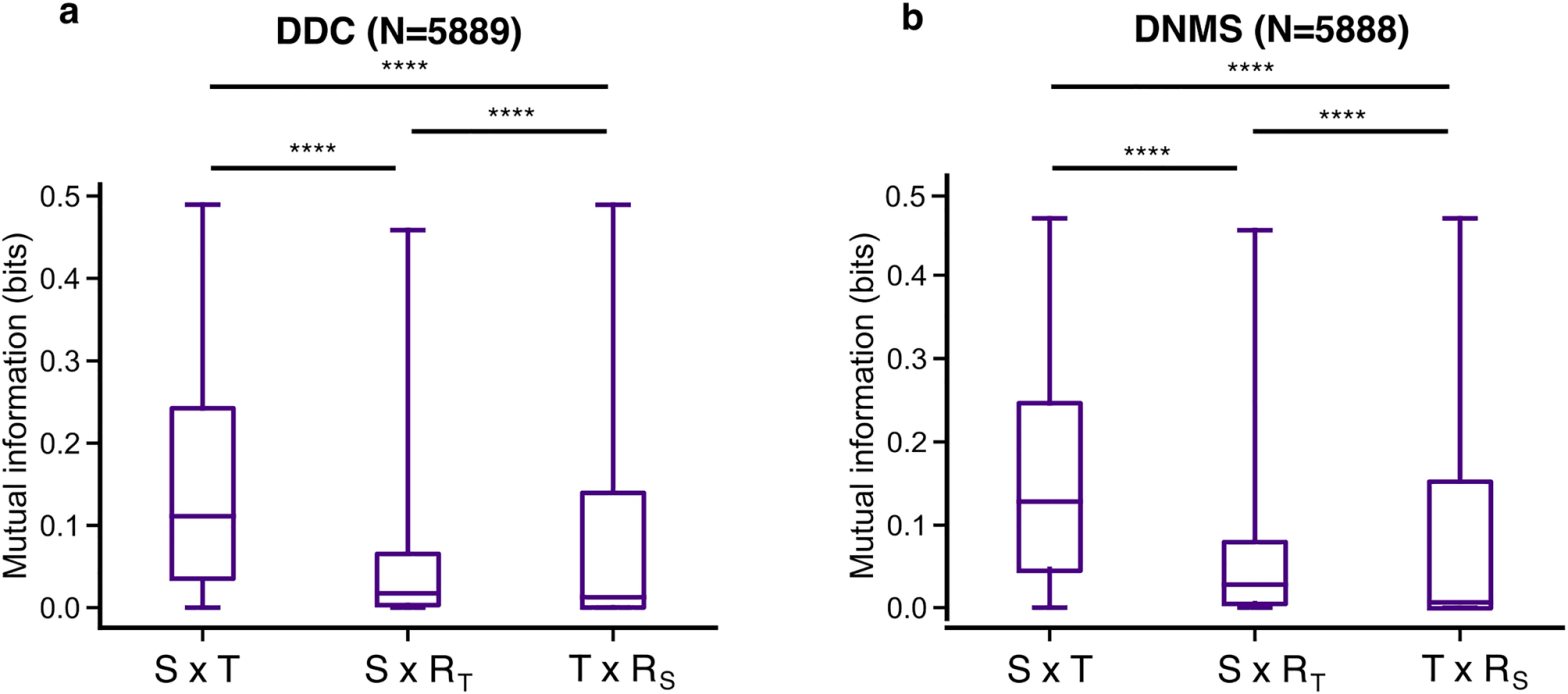
The activity of RNN units carry information about stimulus and elapsed time. (**a**) Boxplots show the distribution of mutual information between the RNN activity during stimulus presentation in the DDC task and occupancy in a two-dimensional joint stimulus-by-time ($S\times T$) space (left column), compared to the amount of mutual information if the units encoded just stimulus (middle column, $S\times {R}_{T}$) or just time (right column, $T\times {R}_{S}$) independently. ${R}_{T}$ and ${R}_{S}$ indicate that the time and stimulus dimension is randomized, respectively. Results were aggregated across 50 agents. Only units with significant mutual information in the non-randomized $S\times T$ space were included in the statistical analysis. RNN activity significantly encoded both stimulus (****Kruskal–Wallis test, N = 5889, p < 0.00001) and time (****Kruskal–Wallis test, N = 5889, p < 0.00001). (**b**) Same as (**a**), but for the DNMS task during the delay period. RNN activity significantly encoded both stimulus (****Kruskal–Wallis test, N = 5888, p < 0.00001) and time (****Kruskal–Wallis test, N = 5888, p < 0.00001). P-values were corrected for multiple comparisons with Bonferroni.

To better understand how time cells and ramping cells may or may not be contributing to temporal calculations in the DRL agents, we next conducted a set of information theoretic analyses and lesion studies. Using tools for estimating mutual information in neural circuits^46^, we calculated the number of bits carried by the recurrent networks about both timing and stimulus during the delay period, in both the DDC and DNMS tasks. As well, we examined how the mutual information estimates were affected by shuffling either the temporal variables, or the stimulus variables. This allowed us to determine how much the joint distribution mutual information was related to either time or stimulus identity separately. We found that for either task the RNNs carried roughly the same amount of information about time and stimulus identity. As well, the shuffling analyses showed that the networks carried information about both time and stimulus identity in each task, as shuffling either variable led to a statistically significant drop in mutual information (Fig. 4).

The information theoretic analysis suggested that the activity dynamics in the networks are indeed encoding information about time and stimulus identity, but whether this information is being used to solve the task directly cannot be determined by such analyses. To determine this, we then turned to a virtual lesion study. Leveraging the manipulability of artificial neural networks, we designed two types of virtual “knock-out" experiments that took advantage of our ability to selectively manipulate activity in specific units and specific times of the RNNs.

**Figure 5:**
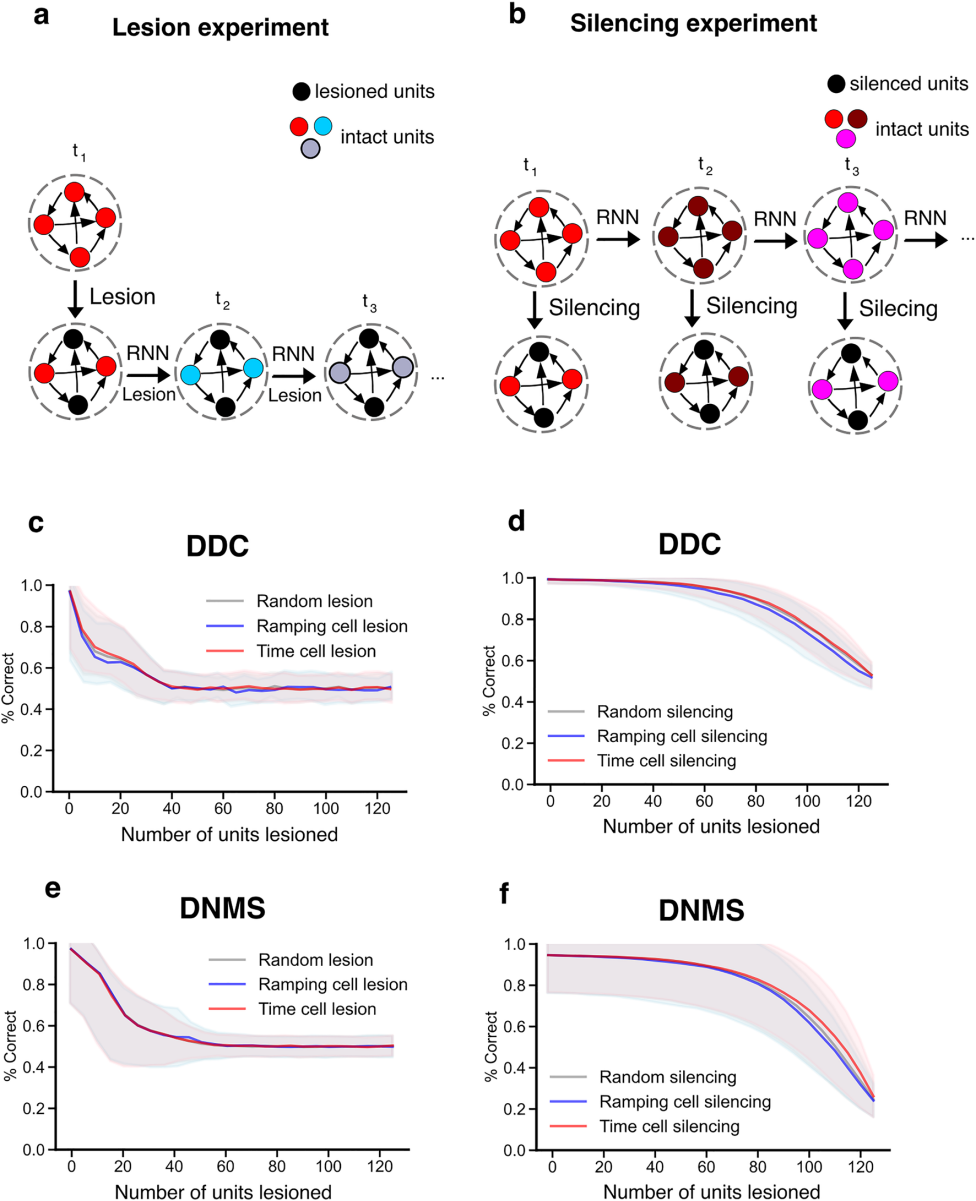
Performance drop in the neural networks with units selectively lesioned or silenced. (**a**) Graphical illustration of the lesion experiment. In these experiments, the targeted units are also governed by the RNN equations just like the non-lesioned units, and therefore, will affect the activity of the rest of the non-lesioned population. (**b**) Graphical illustration of the silencing experiment. In these experiments, the activity of the targeted units is manually fixed to zero while the activity in the non-targeted recurrent population is kept the same as it would be under the normal conditions (top row, governed by the RNN equations). In both (**a**) and (**b**), black circles indicate targeted units with zero activity, while colored circles indicate non-targeted units. Changes in the color of the circle between two consecutive time steps indicate changes in the population activity in the RNN. For visual clarity, differences in the activity of individual units at a given timestep are not reflected in the color of the circles. (**c**–**f**) Percentage of correct responses in 100 trials during which different numbers of RNN units were lesioned in agents trained on the DDC task (**c**) or DNMS task (**d**), or silenced in agents trained on the DDC tasks (**e**) or DNMS task (**f**). Solid lines and shaded areas indicate average and standard deviation across 50 random selections of each cell type per agent, for 50 agents.

In the first type of knock-out experiment, which we termed “lesion experiments”, we set the targeted recurrent unit’s activity to zero at each time step throughout the simulation (Fig. 5a), akin to killing a cell in a neuroscience experiment. We did this “lesioning” after training on the tasks. Notably, as in real lesion experiments, this would affect the activity of the rest of the non-lesioned population, thanks to the recurrent dynamics. As such, any drop in performance in the tasks could be a result of the impaired dynamic representation that arises from value estimation, rather than a result of time cell or ramping cell activity directly driving the downstream policy or value units.

To examine the potential direct role of time and ramping cells in the final computation of policy and value, we also ran a second type of knock-out experiment, which we termed “silencing experiments”. In these experiments, we first recorded the activity in the RNN under normal conditions. Then, at each time step during the experiment, we manually fed in the previously recorded neural activity patterns but with the activity of a targeted set of neurons fixed to zero, i.e., “silencing” them (Fig. 5b). By manually inserting the activity from a normal run like this, we kept the activity in the non-targeted recurrent population the same as it would be without the silencing. Thus, in these experiments the “knock-out” only altered the activity of the targeted neurons, and not the rest of the population, allowing us to isolate the impact of these cells in the downstream policy and value calculations during task performance separately from their impact on the dynamic representation on which policy can be computed.

In the lesion experiments, we found that in both DDC and DNMS tasks, lesioning time cells, ramping cells, or random cells led to equivalent drops in performance (Fig. 5c,e). In all three conditions, the performance on the task dropped as the number of units lesioned increased, and the performance approached chance level asymptotically once the number of units lesioned exceeded a certain amount. This suggests that time cells and ramping cells are contributing to the tasks equally in both the DDC and DNMS tasks.

In the silencing experiments, we again found similar results for the DDC and DNMS tasks, with lesions of time cells, ramping cells, or random cells producing similar drops in performance (Fig. 5d,f). However, unlike in the lesion experiments, we found that many more units had to be silenced to induce a drop in performance. This shows that the contribution of time and ramping cells to the tasks was in large part a result of their impact on the dynamic regime that is needed for policy computation. As well, this implies that the policy and value calculations rely on a highly distributed code for their calculations, one that depends equally on time and ramping cells in both tasks. Thus, altogether, these results demonstrate that time and ramping cells are contributing a great deal to task performance courtesy of their impact on recurrent dynamics. As such, the exact manner in which encoded information about time and stimulus identity is being used is complex, and not a simple function of a small number of units individually contributing a great deal to any temporal or stimulus computations made by the policy or value units. Contrary to the currently widespread belief, our results suggest that units tuned to elapsed time may not be the mechanism the brain uses to read-out “what happened when”, per se, even though they contain information about time, and represent time in a human-interpretable fashion. Instead, they may contribute to temporal calculations as part of the dynamic regime in the brain that is associated with learning the value function.

### Encoding of time is dissociable from behavior in the DDC task

A previous study has suggested that the encoding of time by ramping cells in the rat striatum might be distinct from the perception of time, as measured by behavior in the DDC task^29^. Hence, we sought to determine whether the same holds true in our DRL agents. We found that, during stimulus presentation, the vast majority of the units were tuned globally to the most prolonged stimulus duration and did not rescale across different stimulus duration (example cells shown in Fig. 2c, tuning curves of different colors indicate different durations). This meant that individual units maintained their temporal receptive field regardless of the actual stimulus duration, implying that the recurrent units track the absolute passage of time independent of the behavior that different stimulus lengths could imply.

**Figure 6:**
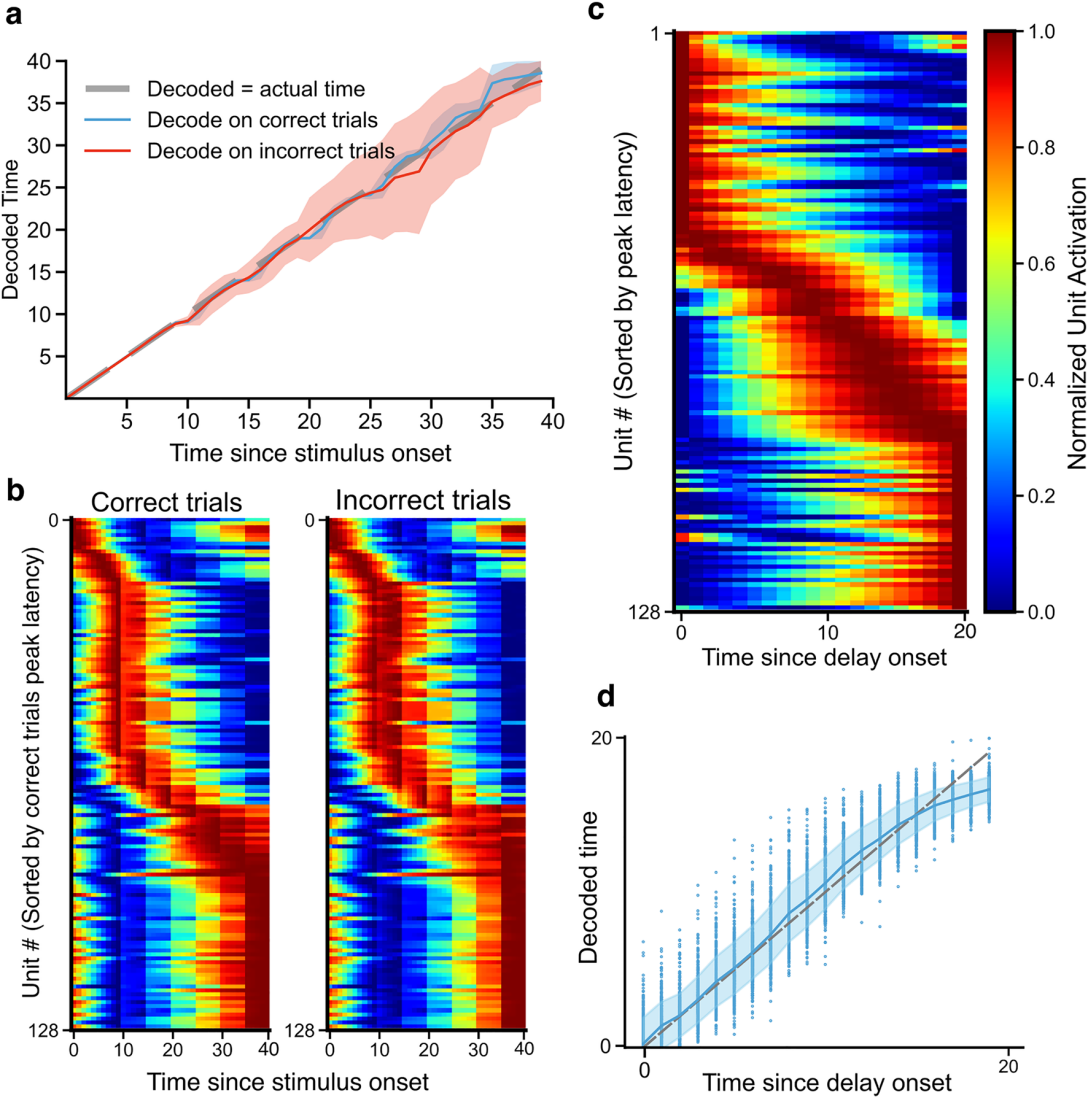
Dissociation between the encoding of time and the behavior of timing in the DDC task. (**a**) Decoding time on either correct (blue) or incorrect (red) trials, using a linear regression decoder trained only on correct trials, plotted against the actual elapsed time. Dashed gray line represents correct decoding. Solid lines and shaded areas indicate average and standard deviation across 5 cross-validation folds per agent for 50 agents. (**b**) The population activity averaged across all stimulus durations for correct trials versus incorrect trials. Rows in both panels are sorted according to the latency to the unit’s peak activity during the correct trials. (**c**) Similar to Fig. 2a, but for the delay period in the DDC task. (**d**) Linear regression decoding of elapsed time since delay onset from the activity of all RNN units at each time step from an example agent. Each dot represents the decoding accuracy of a population vector at one time step on one trial. Blue line and shaded area represent mean and standard deviation of decoded time for each actual time, gray dashed line represents correct decoding.

We therefore examined time encoding and population activity separately for correct and incorrect trials, as done in the previous study in the striatum^29^. If behavior is indeed dependent on the encoding of time by time and ramping cells, then we should observe more errors in temporal decoding for incorrect over correct trials. However, a linear regression decoder predicted the time elapsed with equal accuracy in both the correct trials and the incorrect trials (Fig. 6a), even though the decoder was trained only on the correct trials. This suggests that the temporal encoding provided by time cells and ramping cells did not determine which trials were correct versus incorrect. In other words, there was a dissociation between the temporal information carried by the population activity of time and ramping cells and the agent’s perceptual decision regarding the relative duration of sensory stimuli.

Supporting this, the temporal dynamics in the recurrent population did not differ qualitatively between correct and incorrect trials (Fig. 6b). As well, we observed time cells and ramping cells during the delay period in the task, when no temporal information is required (Fig. 6c). Plus, during the delay period, time elapsed could be accurately decoded by a linear regression decoder from the population activity at each time step (Fig. 6d). Altogether, this suggests that time encoding may emerge as an intrinsic circuit property of RNNs irrespective of the downstream behavior.

### Encoding of stimuli depends on mnemonic demands

**Figure 7:**
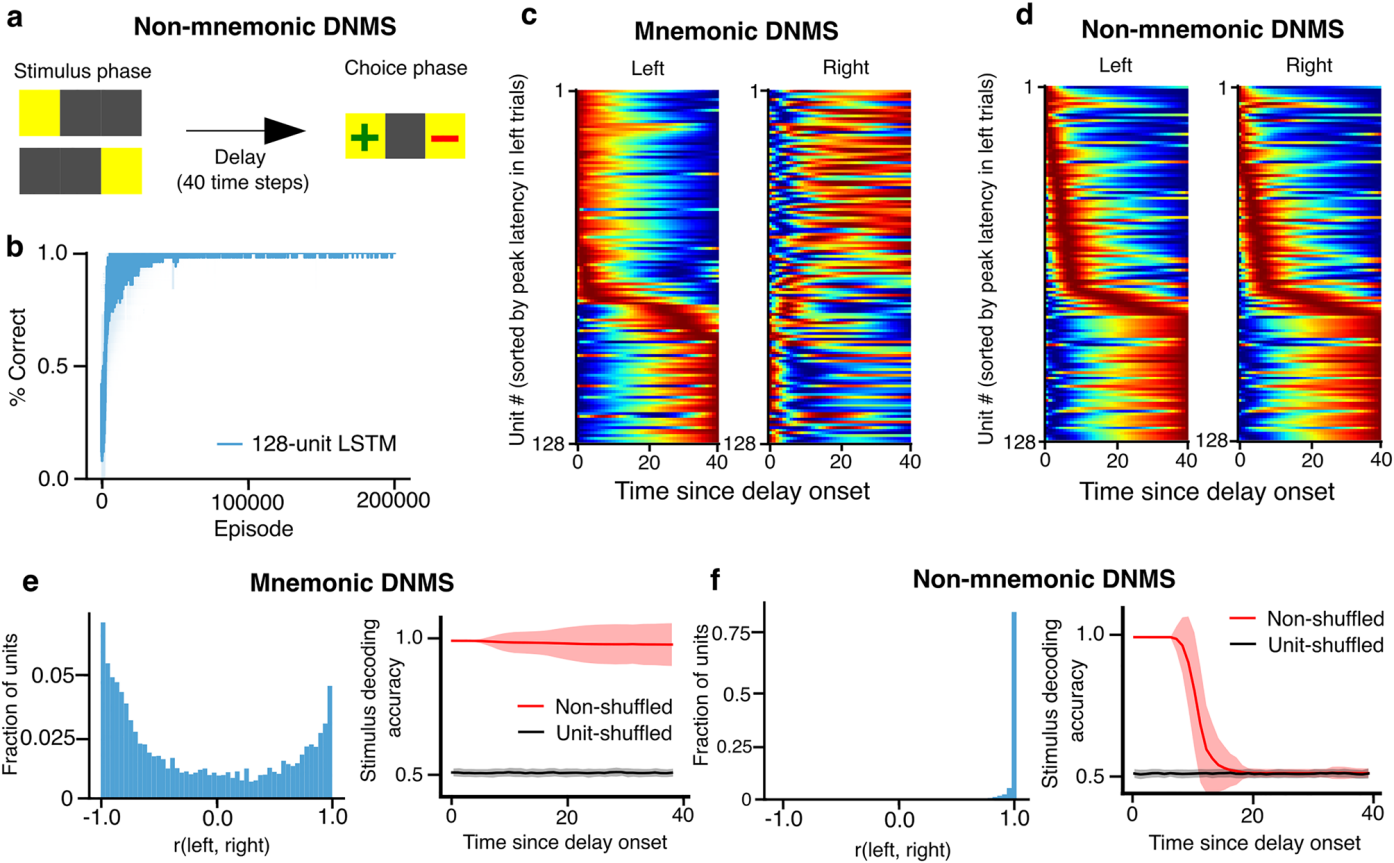
Time cell encoding of sensory stimulus depends on the mnemonic demands. (**a**) Task schema for the non-mnemonic DNMS task. After the delay, the agent must choose the left location regardless of the stimulus location to receive a reward. (**b**) The percentage of correct (i.e., left) choices over the course of training. (**c**, **d**) Ensemble neural activity during the delay period for left- or right-stimulus trials under the normal (**c**) or non-mnemonic (**d**) condition of the DNMS task. Each heatmap shows the trial-averaged hidden state activity of all 128 RNN units, normalized to each unit’s minimum (blue) and maximum (red) activity throughout the recorded episodes. In all heatmaps, the units (i.e., rows) are sorted by the latency to peak activity during the left stimulus trials under the corresponding task condition. (**e**) Left panel: Distributions of Pearson correlations between each RNN unit’s temporal tuning curves for left trials versus right trials during the mnemonic DNMS task for all units in 50 agents. Right panel: SVM decoding of the stimulus displayed prior to the delay period from the population activity at each time step during the delay period of the mnemonic DNMS task. Decoding accuracy is measured by the fraction of test trials decoded correctly. Decoding accuracies from unit-shuffled population activities are plotted in gray and serve as a chance baseline. Solid lines and shaded areas indicate the average and standard deviation across 5 cross-validation folds per agent for 50 agents. (**f**) Same as (**e**), but for the non-mnemonic DNMS task.

Previous research on rodent hippocampal time cells has been inconclusive as to whether these sequential activity patterns indeed contribute to stimulus-encoding in memory or not^8,9,40^. Here, we investigated the effect of mnemonic demand on the temporal representations by developing a non-mnemonic version of the DNMS task. In this version, upon the onset of the choice phase, the agent must choose the left location to receive a reward regardless of the sample location, thus eliminating the demand for the agent to remember the sample location across the delay period (Fig. 7a). The agents trained on the non-mnemonic DNMS task had the same architectures as the single pathway models trained on the normal DNMS task (Fig. 1b). As expected, the agents learned the non-mnemonic task much faster than its mnemonic counterpart (Fig. 7b). We also conducted lesion and silencing experiments on agents trained on the non-mnemonic DNMS tasks and observed that solving the non-mnemonic DNMS task did not rely on recurrence as much as the mnemonic task, suggested by the preserved performance in the lesion experiment (Fig. S5).

To characterize the effect of memory demand, we first examined the temporal organization of recurrent units during different trial types (left sample versus right sample) under mnemonic or non-mnemonic conditions. We found that under the mnemonic condition, the identity of the sensory stimuli were represented by time and ramping cells in the recurrent dynamics throughout the delay, as suggested by the different orders of sequential activation of the neural ensemble during the delay period (Fig. 7c). When we examined each unit’s temporal tuning curves during the delay period following different stimuli, we found that most units had almost orthogonal representations for the two stimuli as suggested by the negative Pearson correlation (Fig. 7e, left). In contrast, in the non-mnemonic task, different sensory stimuli were represented by almost identical neural dynamics (Fig. 7d) and highly correlated temporal tuning curves (Fig. 7f, left), suggesting a lack of representation for the stimuli when it is task-irrelevant.

To quantify how informative these patterns were, we trained a support vector machine (SVM) to decode the identity of the stimulus from the activity of the recurrent units at each time step during the delay period. We found that, when the agent was required to remember the identity of the sample (i.e., mnemonic DNMS), the recurrent units successfully preserved the information about the stimulus in their activity across the entire delay. To confirm the significance of this, we also conducted the decoding using shuffled activities (i.e., the cell identities were shuffled), which led to chance decoding performance (Fig. 7e, right). In contrast, in the absence of working memory demand (i.e., non-mnemonic DNMS), the activity of recurrent units gradually lost information about the stimulus over the course of the delay period eventually settling at chance level (Fig. 7f, right). Thus, our results support the idea that time cell representations can contribute to a lasting record of sensory data in the presence of mnemonic demands.

### DRL agents trained on a spatial working memory task exhibited conjunctive coding of time, space, and stimulus

We had found time is encoded by DRL agents trained on the DNMS task, likely due to the temporal calculations required for value estimation (Fig. 3b). One possible reason for this is that our models receive no inputs over the delay period, and so, dynamic computations (such as estimating value) over the delay period necessarily also carry temporal information. If, however, there were other variables that could change over the delay period, then temporal encoding may be less important. In other words, there may be no disentanglement of temporal representations from representations of other variables when other variables are available during the delay.

**Figure 8:**
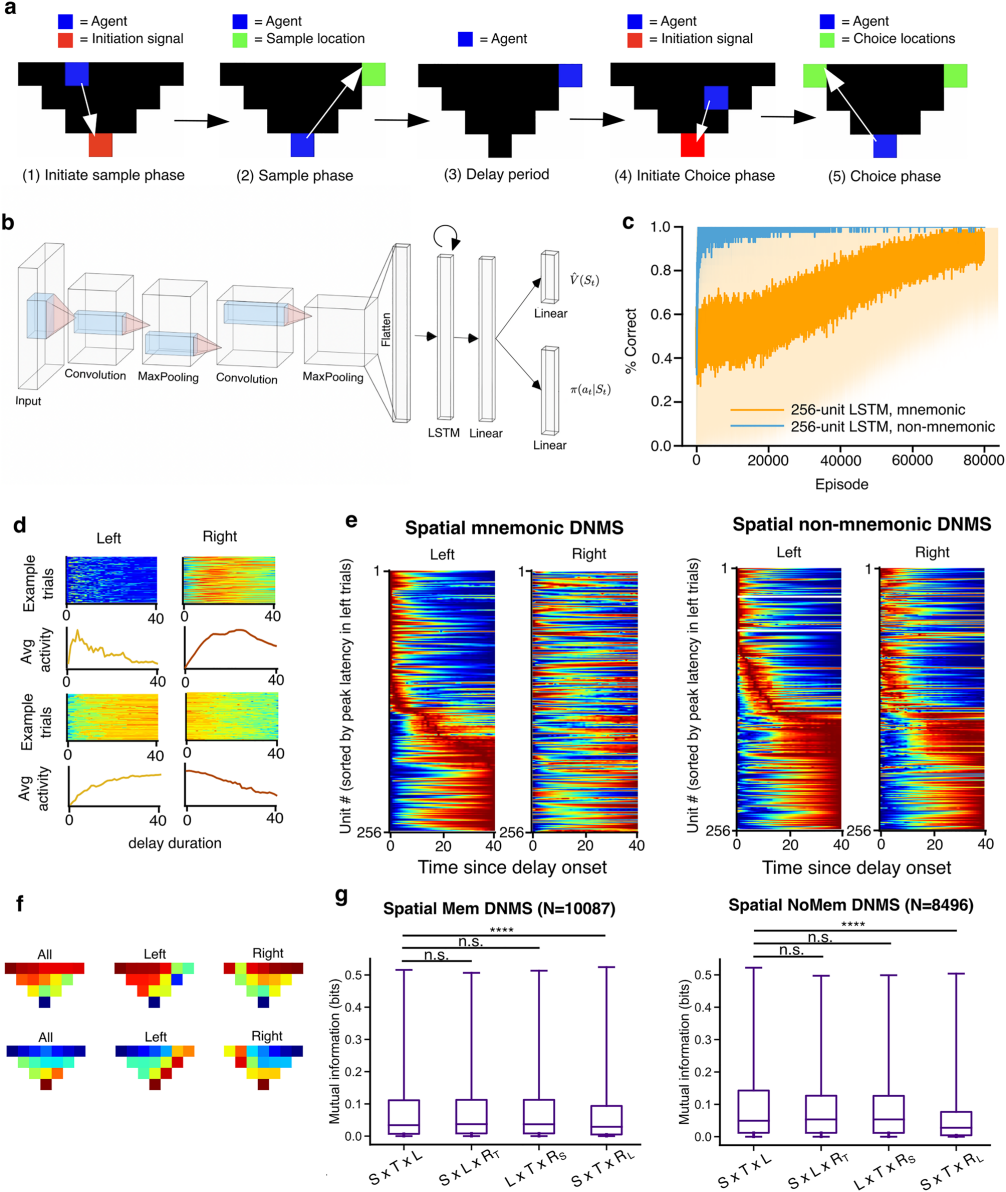
DRL agents trained on a spatial working memory task exhibit conjunctive coding of time, space, and stimulus. (**a**) Schematic illustration of the task structure in one trial of spatial DNMS task. (**b**) Architecture of the DRL agent trained on the spatial DNMS task. (**c**) Performance of the agent on spatial DNMS (orange) and non-mnemonic DNMS (blue) tasks over the course of training, measured by the percentage of choices that led to reward. Solid line and shaded area represent the average and standard deviation of performance over 50 seeds, respectively. (**d**) Similar to Fig. 2d, but for the spatial DNMS tasks. (**e**) Similar to Fig. 6c,d, but for the spatial DNMS tasks. (**f**) Two example neurons that exhibited stimulus-modulated location tuning. Each heatmap shows the occupancy-normalized trial-averaged activity at different locations in all trials (left column), left-stimulus trials (middle column), or right-stimulus trials (right columns). (**g**) *Left panel*: Boxplots show the distribution of mutual information between the RNN activity during the delay period of the spatial DNMS task and occupancy in a three-dimensional stimulus-by-time-by-location ($S\times T\times L$) space (left column), compared to the amount of mutual information if the units only encoded two of the variables: (from left to right) stimulus and location ($S\times L\times {R}_{T}$), time and location ($L\times T\times {R}_{S}$), stimulus and time ($S\times T\times {R}_{L}$), with the third variable randomized. Results were aggregated across 50 agents. Only units with significant mutual information in the non-randomized $S\times T\times L$ space were included in the statistical analysis. RNN activity only significantly encoded location (****Kruskal–Wallis test, p = 0.00001), but not time (n.s., Kruskal–Wallis test, p = 0.117) or stimulus (n.s., Kruskal–Wallis test, p = 0.144). Right panel: same as the left panel, but for the non-mnemonic version. RNN activity only significantly encoded location (****Kruskal–Wallis test, p = 0.00001), but not time (n.s., Kruskal–Wallis test, p = 0.468) or stimulus (n.s., Kruskal–Wallis test, p = 0.330). P-values were corrected for multiple comparisons with Bonferroni.

To this end, we altered the DNMS task by making a version in a triangular arena, similar to the arenas used with animals^47^. In this scenario, the agents can freely move, even during the delay period (“spatial DNMS task”; Fig. 8a), and as such, space becomes another variable that can change over the delay. All components of the task were the same, except that the two stimuli are placed at the left and right sides of the arena, and the initiation signal is placed at the bottom of the arena. The agent must navigate to these locations and interact with them to proceed to the next stage of the task. To maximize the reward, the agent must not only remember the stimulus presented, but also navigate to desired locations in the shortest path possible without taking redundant actions. The agent used in the spatial DNMS task was similar to the one shown in Fig. 1b, but we added a convolutional neural network to generate a latent representation of the visual input, an RGB image of the environment from above (Fig. 8b). We also simulated the non-mnemonic version of the spatial DNMS task in which, after the delay, the agent must navigate to and interact with the left location to receive reward regardless of the sample. As expected, The DRL agents learned both tasks, with the non-mnemonic version learned much faster (Fig. 8c).

We then examined the representations of task-relevant variables by the recurrent units. We found that, similar to the location-fixed DNMS task, the recurrent units during the spatial DNMS task also exhibited trial-reliable temporal tuning, resembling time cells and ramping cells (Fig. 8d). We also found that the recurrent dynamics only differed for the two stimuli when there was a mnemonic demand (Fig. 8e). Plus, we observed spatial tuning, modulated by the sensory stimuli presented before the delay period, similar to the “splitter cell” phenomenon observed in the rodent hippocampus^48,49^ (Fig. 8f). We then conducted mutual information analyses using stimulus, time, and location as variables. We found that the RNNs carried joint information about stimulus, time, and location in the spatial DNMS tasks, but they only carried significant information about spatial location, as this was the only variable whose shuffling led to a significant drop in mutual information (Fig. 8g). This suggests that time cells and ramping cells will appear as a result of natural recurrent dynamics and the timing calculations involved in estimating value, but these cells do not necessarily carry disentangled information from other relevant variables, such as space. In summary, we observed conjunctive coding of space, time, and stimulus in the same recurrent population, which pointed to the possibility that the temporal selectivity of time cells and ramping cells may represent task-relevant variables that correlate with time rather than time, per se.

## Discussion

In this study we used DRL agents to ask whether time and ramping cells necessarily play a direct role in encoding time and memory, or whether they may also be an “epiphenomenon” of recurrent neural network dynamics. We found that time and ramping cells emerge in DRL agents trained on both a task that, while performing the task, requires keeping track of time (DDC) and a task that, while performing the task, requires working memory (DNMS) (Figs. 1 and 2). Notably, the emergence of time and ramping cells in the DNMS task appeared to be driven by the value learning objective (Fig. 3), possibly because value calculations require a temporal estimate. We also found that the recurrent activity in these agents encoded time accurately (Fig. 4). But, the role of these cells in the behavior of the networks was complicated and related at least in part to their contributions to the dynamic regime that arose from learning to estimate the value, on which the policy was computed (we also note that value estimation is needed only when the agent is learning the task since it would reduce the variance in weight updates and facilitate training, but not executing the task, at which point only policy computation would suffice) (Fig. 5). As well, the encoding of time was dissociable from the performance of the networks (Fig. 6). In a task that required only value calculations (non-mnemonic DNMS) time and ramping cells still emerged (Fig. 7). But, when the task involved a spatial dimension, time and ramping cells did not encode time or stimulus separately from spatial location (Fig. 8). In total, these results suggest that time and ramping cells may indeed contribute to encoding of time and memory, but how they do so depends on the nature of the task. Furthermore, our results suggest that the role of these cells could relate to recurrent circuit dynamics. Given this, we believe that neuroscientists should utilize caution when making any interpretation of temporal encoding due to the presence of time or ramping cells alone. One possibility, hinted at by our results, is that these cells are a natural product of any system attempting to estimate value in a recurrent circuit.

Our results using DRL models replicated several findings in the animal brain. For example, the DRL agents trained on the DDC task showed a dissociation between time encoding by ramping cells and time cells and time perception, replicating the observations in the rat striatum by Toso et al.^29^. When trained on the DNMS task, the recurrent units in the DRL agents exhibited sequential activation patterns that tiled the delay period with or without a memory demand, replicating the observations in the rat hippocampus by Salz et al.^9^. When a memory demand is present, the time cells in the DRL agent demonstrated distinct temporal tuning during the delay period, replicating the observations in the rat hippocampus CA1 by MacDonald et al.^36^. Importantly, our DRL models, trained only to optimize task reward, capture some of the properties of time cells that we did not train them on at all, such as an increase in the width of the temporal receptive field later in the interval^5,6,9,12,15,17,18,39^. As such, our DRL models provide a normative and algorithmic substrate for understanding the ubiquity of time cells and ramping cells in the brain, and they can help us to integrate multiple findings across different brain circuits.

There are several limitations of our task-optimized DRL model. First, our model was not a physiological model, since it utilized non-physiological recurrent dynamics with an assumption of rate-based coding (see “Methods”) and was trained with backpropagation through time. Second, our DRL agents required tens of thousands of training episodes, which was much more than what is usually needed for animals to learn the same task. But, we note that our agents are trained from scratch, thus do not have the priors animals have, provided by their innate wiring and the experience through their lifetimes. Third, our study used toy simulations of real experiments used in the neuroscience literature, which calls for simulation experiments in more realistic 3D environments to confirm our findings. Fourth, when training our networks we reset the hidden unit activity between trials, which may or may not be an accurate reflection of real experiments, depending on how long of a delay occurs between trials during training. Lastly, while our simulation data was collected after the agents were trained on the tasks, with the weights frozen (i.e., during the inference phase, with no learning occurring), real animals would always be learning and thus constantly updating their value estimations. The fact that we saw fewer ramping cells in the DNMS task when hidden unit activity was not reset (and even observed some oscillatory units Fig. S3) suggests that the length of delay between trials may be another experimental factor to consider when looking for ramping cells in real experiments. Lastly, we note that in our study, even though we used the same criteria to classify time cells and ramping cells as the animal studies, we observed a much higher percentage of time cells and ramping cells in the recurrent population, likely because real neurons are much noisier and more likely to be silent during the experiment than artificial units. Another potential explanation is that real animals have learned many more things than a single task (or pair of tasks). It is possible, therefore, that many neurons in real brains are simply tuned to task-unrelated information, reducing the number of observed time and ramping cells.

In the past several decades, neuroscientists have discovered neurons tuned to a plethora of different variables in a wide range of species^50–53^. However, our results suggest that one must be cautious when associating the variables these neurons are tuned to with their functions. In this case, ramping cells and time cells are indeed tuned to elapsed time, but this is because they are defined by the change in their firing rate over time, and identified via analyses that presume their tuning properties. One example of such analyses is sorting neurons based on the latency to their peak activity, which is bound to result in a sequence due to the dynamical nature of recurrent neural circuits. Some may rebut by saying that time cells and ramping cells are not just tuned to time; they carry information about time. However, our results on info-theoretic analysis and knock-out experiments suggest that carrying information about a variable does not equate to helping the brain manipulate information about this variable to accomplish cognitive tasks, necessarily; as we have seen, time could simply be a correlated variable in the neural representation of other task-relevant variables, such as space (Fig. 7g, right). Instead, we argue that what is important is how downstream networks parse and use information carried by these neurons tuned to particular variables, including time. Lastly, some may argue that, it is now common practice in animal experiments on time cells and ramping cells to hold every other variable (e.g., head direction, location, distance) constant to make sure that time cells and ramping cells only represent time. However, we observed that apparent time cells and ramping cells emerged even when there are no other variables that are being tracked by the network other than value, and no need to track time (e.g., the delay period during the non-mnemonic DNMS task, or the delay period of the DDC task), showing that these cells are a natural emergent property of almost any recurrent neural network trained to estimate value over time. In line with this point, studies on time cells in the rodent hippocampus, which constitute the majority of the time cell literature so far, suffer from an inevitable dilemma: running or movement of the animal leads to higher firing rate of hippocampal neurons^54,55^. Most time cell studies fix the animal’s location by having the animal running on a treadmill; in the absence of such behavior that unfolds over time, the hippocampal network is less likely to show time cells^8^. In sum, our results show that caution must be used when interpreting data on time cells and ramping cells, and these neurons could reflect changes in neural dynamics and value-based learning, rather than time tracking required when performing the task, per se. After all, without any changes, how do we really know that time has elapsed?

## Methods

### Simulated cognitive task environments

The simulated environments for the DDC and DNMS tasks were designed to be compatible with the OpenAI gym framework^56^, a suite of environments with which reinforcement learning agents interact in discrete time steps. In non-spatial environments, the states (observations) of the environment were one-hot encoded. In the DDC task, in each episode, the agent received two stimuli of varying length presented sequentially, whose durations were sampled uniformly among 10, 15, 20, 25, 30, 35, and 40 time-steps, separated by a 20 time-step delay period. The two stimuli must have different durations. The agent had two possible actions: to indicate whether the first or the second stimulus was longer. Depending on the ground truth, the agent would either receive a scalar reward (+ 1) or punishment (− 1) and the episode would conclude. In the DNMS task, in each episode, the agent received one of two possible stimuli (“left” or “right”), and had to interact with the stimulus to proceed to a delay period of 40 time-steps. After the delay period, the two stimuli were simultaneously presented to the agent, and the agent had to choose the stimulus that was different from the one it received to receive a reward and finish the trial. In the non-mnemonic version of the DNMS task, after the delay period, the agent had to choose the “left” stimulus to receive a reward and finish the trial, regardless of what stimulus was presented before the delay period, thus eliminating the working memory demand during the delay period.

In spatial DNMS tasks (mnemonic and non-mnemonic), the environment consisted of an inverted-triangular grid arena in which the agent could freely move, surrounded by walls of at least 1 grid thick to make up for a 4-pixel by 7-pixel rectangular visual field input. The state of the environment was rendered as a colored image, with the initiation signal in red, the left and right sample signals in green, the agent in blue, available grids in black, and the walls in white, spatially arranged similar to the arenas used with animals^47^. The agent had six possible actions: move up, move down, move left, move right, interact with the signal at its current location, or stay at the same location without doing anything. When presented with a signal, the agent not only had to move to the signal location but also interact in order to proceed in the trial. To obtain a reward (+ 100), the agent had to select stimuli as in the non-spatial versions of the task. To teach the agent to move to the goal location in the shortest path possible, all actions (except for interacting with the signal when appropriate) were punished slightly (− 5). The structure of each trial mirrored its non-spatial counterpart as described above.

### DRL agent architectures and training details

The DRL agents used to solve tasks in non-spatial environments consisted of a memory module and an actor-critic module. The memory module consisted of an RNN layer with 128 units and a linear layer with 128 units. For the RNN layer, we used long short-term memory (LSTM) modules. Though not as physiological as vanilla RNNs, they help avoid vanishing and exploding gradient issues in vanilla RNNs, thanks to their gating mechanism and linear cell^57^. The output of the linear layer was then fed forward to the actor-critic module, which consisted of a value network that generated an estimate of state value $\widehat{V}({S}_{t} , \theta )$ and a policy network that generated a stochastic policy $\pi ({a}_{t}|{S}_{t} , \theta )$ from which the action would be sampled using a softmax distribution with temperature of 1. For the spatial environments, the DRL agents additionally had a visual module, which is a deep convolutional neural network to generate a latent representation of the image input of the environment state. The convolutional neural network consisted of two convolutional blocks with feature map counts of 16 and 32, respectively. Each block had a convolutional layer with kernel size 2 × 2 followed by max pooling with kernel size 2 × 2 and stride 1 × 1. The output of the visual module was passed to the memory module, which consisted of an LSTM layer with 256 units and a linear layer with 256 units, and then the actor-critic module.

The hidden states of the LSTM units were initialized to 0 at the beginning of training. After finishing each trial, the hidden states of the LSTM units were reset to 0 to prevent the hidden state activity from growing too large or dissipating. We conducted a control experiment in which we never reset the hidden units in the network between trials (Fig. S3). To do this, we made a copy of the value of hidden states at the end of each trial, and initialized the hidden states to the copied values.

Our agents were trained with the Asynchronous Advantage Actor-Critic (A3C) algorithm^58^, in which the network parameters were adjusted to minimize the loss $L = {L}_{\pi }+ {L}_{V}$, where

$$L_{\pi } = \sum\limits_{{t = 0}}^{{T - 1}} { - log\pi *(R_{t} - \hat{V})}$$

$$L_{V} = \sum\limits_{{t = 0}}^{{T - 1}} {l_{1} (\hat{V},R_{t} )}$$

where $t = 0, 1, \ldots, T-1$ index the time steps in an episode with $T$ environment steps, ${R}_{t}={\sum }_{i=0}^{t}{\gamma }^{i}{r}_{t-i}$ denotes the discounted return at $t$ calculated from all previous rewards ${r}_{0}, ..., {r}_{t}$, and ${l}_{1}$ is the smooth L1 loss. For all tasks, we used a discount factor $\gamma =0.99$.

The separate actor-critic networks had two separate pathways to estimate value and generate policy, respectively (Fig. 3a). The input to each pathway is the current observation ${S}_{t}$, then passed through each pathway’s memory module (an LSTM layer with 128 units, then a linear layer with 128 units). Then, for the policy pathway, the output from the memory module is passed to a linear layer with the same number of units as the number of actions to generate policy $\pi ({a}_{t}|{S}_{t} , \theta )$, and for the value pathway, the output from the memory module is passed to a linear layer with one unit to calculate the estimated value $\widehat{V}({S}_{t} , \theta )$.

The model parameters were adjusted to descend the loss gradient using Adam^59^ with ${\beta }_{1}=0.9$, ${\beta }_{2}=0.999$, $\epsilon =1{\text{e}}-8$, batch size = 1. We used a learning rate of 1e − 04 for the mnemonic DNMS task, 5e − 05 for the non-mnemonic DNMS task, 1e − 05 for DDC task, 5e − 06 for the mnemonic and non-mnemonic spatial DNMS task. These learning rates were selected by a grid-based hyperparameter search. For all tasks, we trained 50 agents initialized with different random seeds and pooled results across the agents.

For the transfer learning experiments (Fig. S2), we (1) trained agents that were trained on the DDC task on the DNMS task, in which case we used learning rate of 5e − 05 for the subsequent DNMS task and initialized with 50 random seeds, and (2) trained agents that were trained on the DNMS task on the DDC task, in which case we used learning rate of 1e − 05 for the subsequent DDC task and initialized with 50 random seeds. In both cases we increased temperature in the softmax function in the policy layer from 1 to 1.5 to encourage exploration.

### Data analysis

#### Identification of ramping cell and time cell

For all tasks, after the agent's performance had plateaued (> 90% correct; 150,000 episodes for the DDC task, 200,000 episodes for the non-spatial DNMS tasks, 80,000 episodes for the spatial DNMS tasks), we recorded the hidden state activity of the LSTM units in the network during the period of interest (i.e., the stimulus presentation period and the delay period for DDC task, only the delay period for DNMS task) for 5000 episodes, and normalized each unit’s activity according to its maximum and minimum during these 5000 episodes. We then calculated the temporal tuning curves of each unit by averaging its activity during the period of interest across episodes (i.e., trial-averaged activity). Note that our models assume rate-based coding, i.e., each unit has a real-valued activity which is analogous to the firing rate of real neurons in the brain.

The identification of ramping cells and time cells in the present work was based on Shikano et al.^23^. To determine whether a unit was a ramping cell, we fitted a linear regression to the temporal tuning curve of each unit. Units with p ≤ 0.05 and Pearson correlation coefficient ≥ 0.9 were considered candidate ramping cells. To determine whether a unit was a time cell, we calculated its temporal information based on Skaggs et al.^46^:

$$I = \sum\limits_{t} {\lambda (t)log_{2} \frac{{\lambda (t)}}{\lambda }p(t)}$$

where $\lambda (t)$ is the mean activity of the unit in response at time $t$, $p(t)$ is the probability density of being at time $t$, and $\lambda$ is the overall mean activity of the unit. If a unit was classified as a ramping unit, its temporal information was calculated from a tuning curve after subtracting the regression line from the temporal tuning curve. Units with significant temporal information (higher than 99 percentile compared to 100 shuffled tuning curves) were considered candidate time cells. In addition, to ensure that the tuning curves were meaningful, we calculated the trial-reliability score of each unit by computing the Pearson correlation coefficient between the tuning curves obtained from even-numbered trials and odd-numbered trials; candidate ramping cells or time cells had to have a significant trial-reliability score (higher than 99 percentile compared to the scores obtained from 100 pairs of shuffled tuning curves) to be considered actual ramping cells or time cells. Each shuffled tuning curve was obtained by circularly shuffling the activity in each episode by a random number of time steps and then taking the average across episodes.

#### Mutual information

The information-theoretic analysis in the present work was based on Skaggs et al.^46^ but extended to consider multiple variables. Specifically, to calculate the information about stimulus and time carried by the unit, we used the formula

$$I = \sum\limits_{{(s,t)}} {\lambda (s,t)log_{2} \frac{{\lambda (s,t)}}{\lambda }p(s,t)}$$

where $\lambda (s,t)$ is the mean activity of the unit in response to stimulus $s$ at time $t$, $p(s,t)$ is the probability density for the agent receiving stimulus $s$ and being at time $t$, and $\lambda$ is the overall mean activity of the unit. To randomize either stimulus or time, we reconstructed $\lambda (s,t)$ and $p(s,t)$ by resampling a new stimulus ${s}^{\prime}$ or time ${t}^{\prime}$ randomly from the their probability distributions, and substituting $s$ with ${s}^{\prime}$ or $t$ with ${t}^{\prime}$ when calculating $\lambda (s,t)$ and $p(s,t)$. We only included units with significant mutual information in the non-randomized $(s,t)$ space (i.e., more than 2 standard deviations from the mean in the distribution of mutual information obtained from shuffling the activity 100 times) in the final results. Because mutual information is not normally distributed, we used a nonparametric paired difference test, namely the Kruskal–Wallis test, to test for significance; the p-values were corrected for multiple comparisons with Bonferroni.

Similarly, for the spatial DNMS tasks, we calculated the information about stimulus, time, and location carried by the unit with the formula

$$I = \sum\limits_{{(s,t,l)}} {\lambda (s,t,l)log_{2} \frac{{\lambda (s,t,l)}}{\lambda }p(s,t,l)}$$

where $\lambda (s,t,l)$ is the mean activity of the unit in response to stimulus $s$ at time $t$ at location $l$, $p(s,t,l)$ is the probability density for the agent receiving stimulus $s$ and being at time $t$ and at location $l$, and $\lambda$ is the overall mean activity of the unit. To randomize one of the dimensions, we reconstructed $\lambda (s,t,l)$ and $p(s,t,l)$ by resampling a new stimulus ${s}^{\prime}$ or time ${t}^{\prime}$ or location ${l}^{\prime}$ randomly from their probability distributions, and substituting $s$ with ${s}^{\prime}$ or $t$ with ${t}^{\prime}$ or $l$ with ${l}^{\prime}$ when calculating $\lambda (s,t,l)$ and $p(s,t,l)$. We only included units with significant mutual information in the non-randomized $(s,t,l)$ space in the final results.

#### Decoding of stimulus and time from single-time population activities

We used support vector machine decoders to quantitatively assess how well the activity of the LSTM population at a given time during the delay period could predict the sample presented prior to the delay. For each time step, the population activity and sample identity data was split into 5 cross-validation folds of train/test datasets, with one-fold held out as testing data, and the remaining 4 folds as training data. To confirm that information about the stimulus was indeed carried by the order of cells in the population, we shuffled the order of cells in each episode at each time point, and constructed separate decoders to decode the sample identify from shuffled data with the same procedure above. The decoder accuracy was measured by the fraction of test trials for which the sample was decoded correctly.

Due to the continuous nature of time, we used linear regression decoders to assess how well the activity of the LSTM population at a given time point within a period can decode the elapsed time since the beginning of the period. For each task condition and interval duration, we pooled the single-time population activity from all recorded intervals under that task condition and duration, and trained a linear regression decoder on 60% of population activity vectors selected at random from that pool to predict the time elapsed since the onset of the interval from the population activity. The remaining 40% of population activity vectors were used as the testing dataset.

### Lesion and silencing experiments

To prepare for the lesion and silencing experiments, we first randomly selected targeted units in the ramping cell pool, time cell pool, or any cells in the population to be lesioned/silenced. For each cell type lesioned/silenced, we started at randomly selecting 5 LSTM units from the pool to be lesioned/silenced, and gradually increased the number of units lesioned/silenced by a step size of 5, up until 125 units were lesioned. When increasing the number of units lesioned/silenced, we randomly selected 5 more units from the pool to be targeted, in addition to the existing targeted units. When the number of targeted units surpassed the total number of ramping/time cells in this agent, we recruited cells of other types as targets. This process of randomly selecting target units was repeated 50 times per agent per cell type to ensure that the change in performance was not only because certain “very important units” within the targeted units were lesioned.

To lesion an LSTM unit, we set the hidden state and the cell state of the LSTM unit to 0 during the forward pass of the environmental state in the neural network, and selected actions from the policy ${\pi}^{\prime}$ downstream of the lesioned LSTM layer to interact with the environment. To silence an LSTM unit, at each time step, we first passed the environmental state through the neural network under the normal condition, cloned the activity of the LSTM layer, then set the activity of targeted neurons to zero in the cloned activity. We then passed the altered clone to the subsequent linear layers to compute the new policy ${\pi}^{\prime}$ and sampled actions from ${\pi}^{\prime}$. The performance of the network was evaluated on actions sampled from ${\pi}^{\prime}$.

### Supplementary Information

Supplementary Figures.

## Data Availability

The datasets generated during and/or analyzed during the current study are available from the corresponding author on reasonable request.

## Code availability

All experiments were performed using PyTorch^60^. All data were analyzed in Python. Decoding analyses were performed using scikit-learn packages^61^. Additional analyses were performed using custom Python scripts, all of which are available on the author’s GitHub account [https://github.com/linclab/deeprl-timecells].
