# Supplementary material for: Temporal encoding in deep reinforcement learning agents
Source: Sci Rep. 2023 Dec 15;13:22335. doi: 10.1038/s41598-023-49847-y (PMC10724179; doi:10.1038/s41598-023-49847-y)
Supplement: Supplementary file 1 — Supplementary Figures. [file 41598_2023_49847_MOESM1_ESM.pdf]

Supplementary Figures

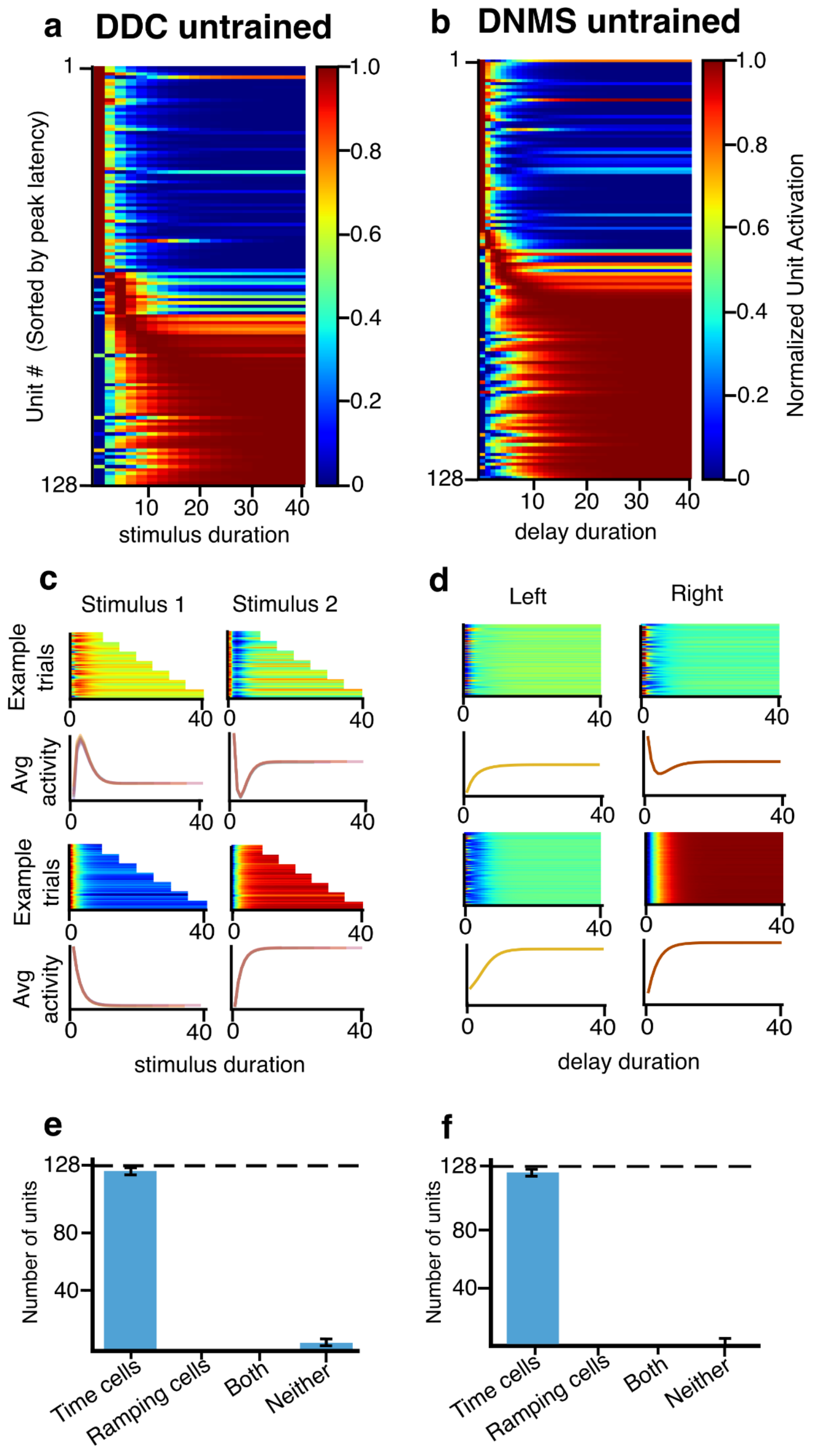

Figure S1: **Ramping cells and stimulus-selectivity are developed over the course of training.** Similar to Fig. 2, but for untrained agents.

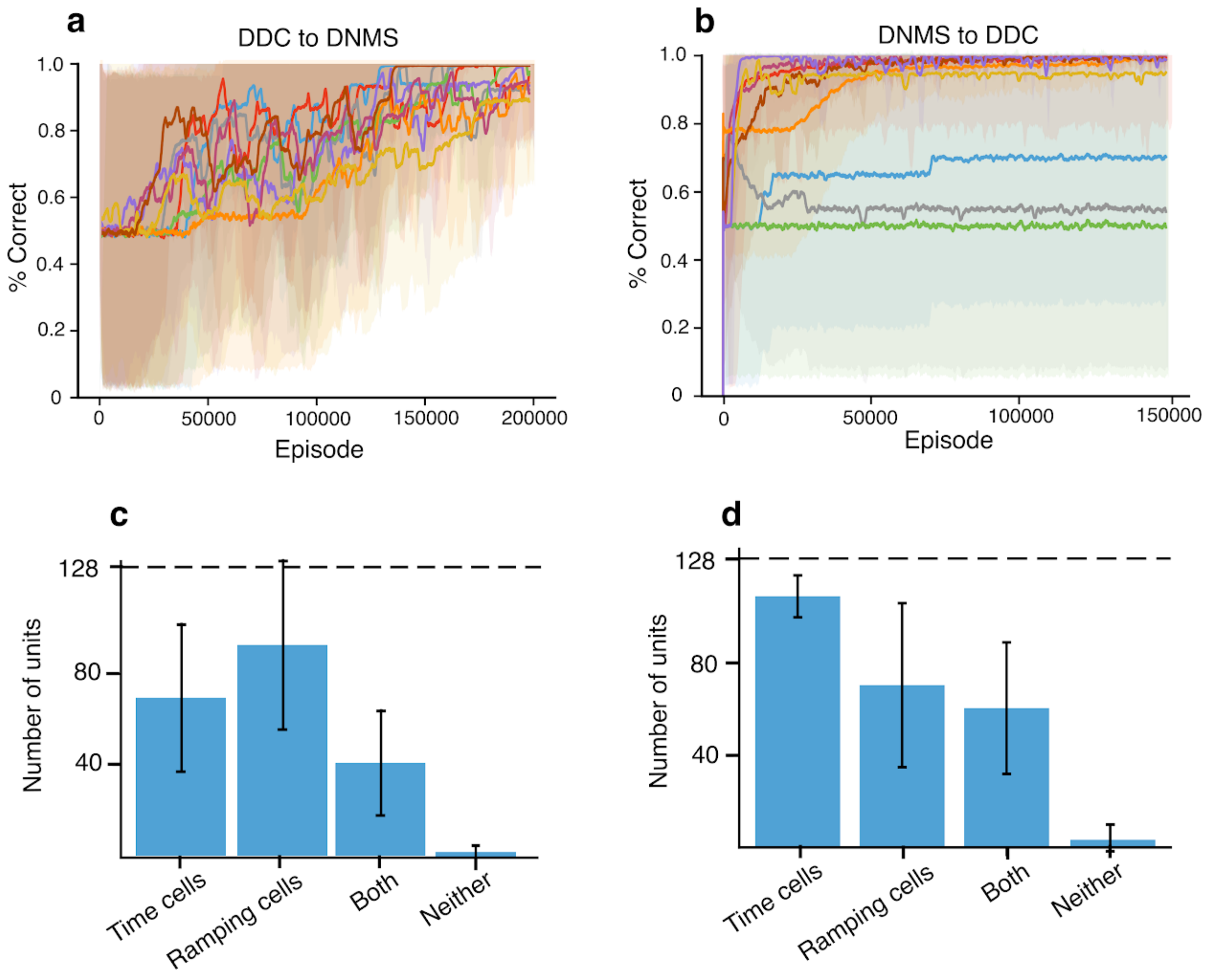

Figure S2: Transfer learning. a) Mean and standard deviation of performance on the DNMS task across 50 random seeds. DIfferent coloured lines indicate different agents pretrained on the DDC task. **b)** Same as a), but for agents pretrained on DNMS then subsequently trained on DDC. **c)** Number of RNN units classified as time cells, ramping cells, neither, or both, in agents pretrained on DDC task and subsequently trained on DNMS task. Error bars indicate standard deviation across all agents. Dashed line indicates the total number of RNN units in the network (i.e. 128). **d)** Same as c), but for agents pretrained on DNMS then subsequently trained on DDC.

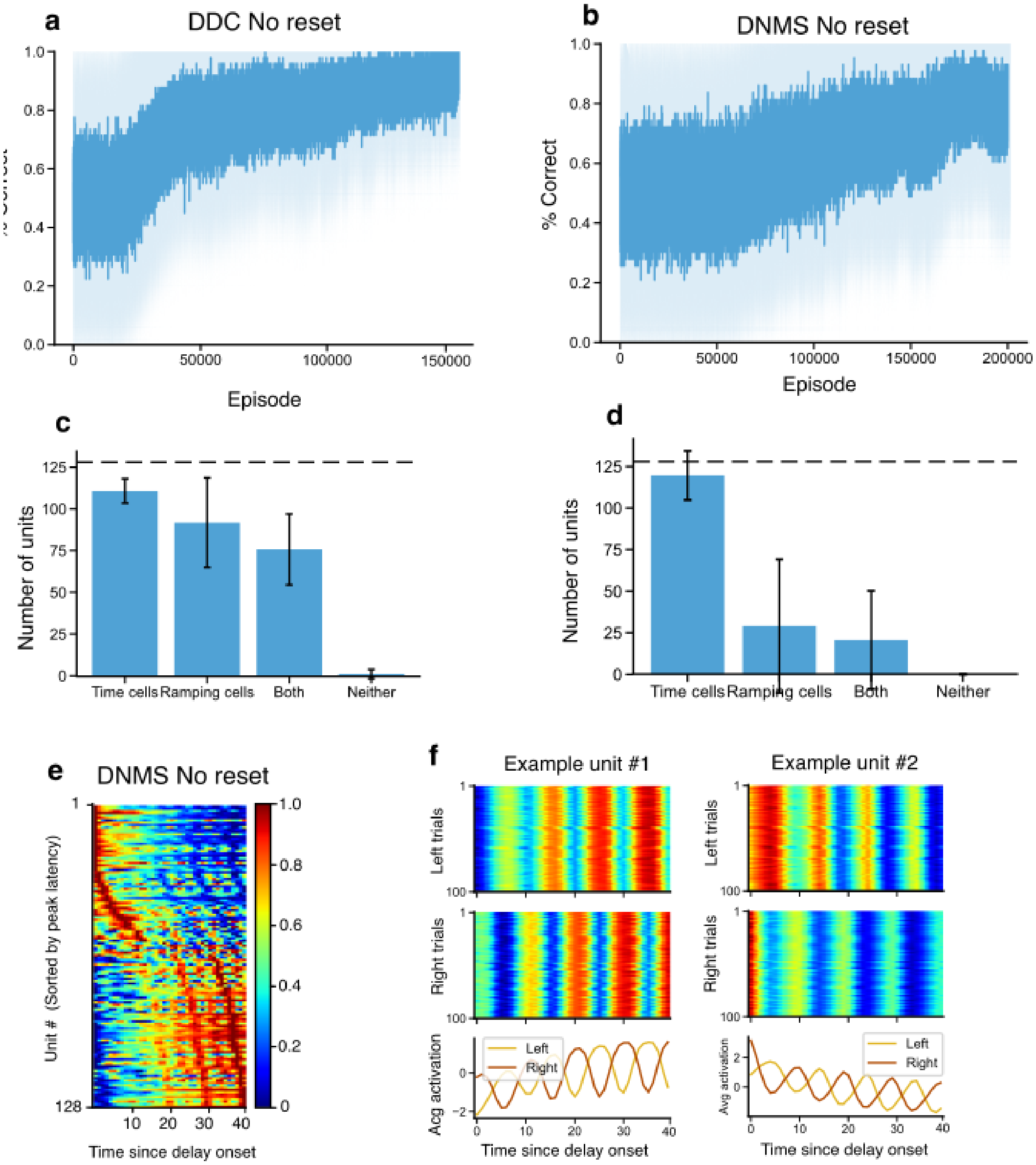

Figure S3: Networks trained on DDC and DNMS tasks without resetting the hidden states. a) The performance of 50 agents without hidden-state reset on the DDC task , measured by the percentage of correct responses. Solid line and shaded area indicate mean and standard deviation across agents, respectively. **b)** Same as a), but for the DNMS task without hidden-state reset. **c)** The number of units qualified as time cell, ramping cell, both time cell and ramping cell, and neither time cell nor ramping cell in each agent averaged across 50 agents trained on the DDC task without hidden-state reset . Error bars indicate the standard deviation. Dashed line indicates the total number of RNN units in the network (i.e. 128). **d)** Same as c), but for the DNMS task without hidden-state reset. **e)** The heatmap shows the RNN activity during the delay period in an example agent trained on the DNMS task without hidden-state reset.

Each row shows the trial-averaged activity of a single unit normalized to its minimum (blue) and maximum (red) activity throughout the recorded episodes. Rows in each panel are sorted by the latency to the peak trial-averaged activity of units. **f)** Two example RNN units from an agent trained on the DNMS task without hidden-state reset. For each unit, the heatmaps show normalized RNN activity during the delay period in 100 consecutive trials for left (top panels) and right sample (middle panels). The bottom panels show the trial-averaged responses during the delay period in all recorded trials, for left trials (yellow curve) and right trials (brown curve).

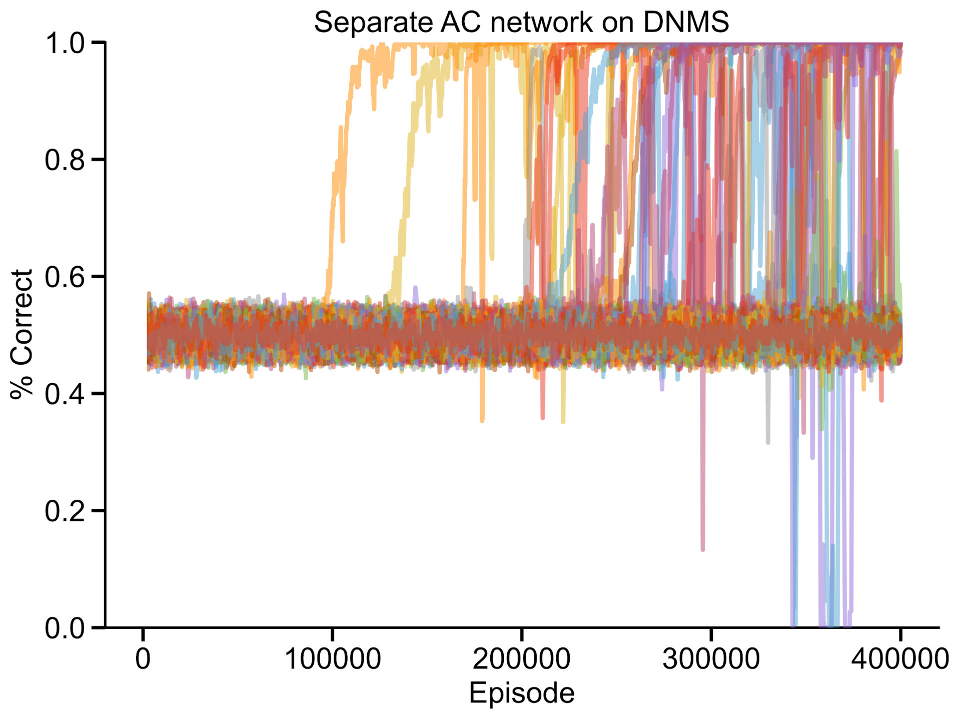

Figure S4: Performance of 70 agents with separate actor (policy) and critic (value) pathways on the DNMS task, measured by the percentage of correct responses. Different agents are initialized with different seeds and plotted in different colors. We collected data from 20 agents that successfully learned the task.

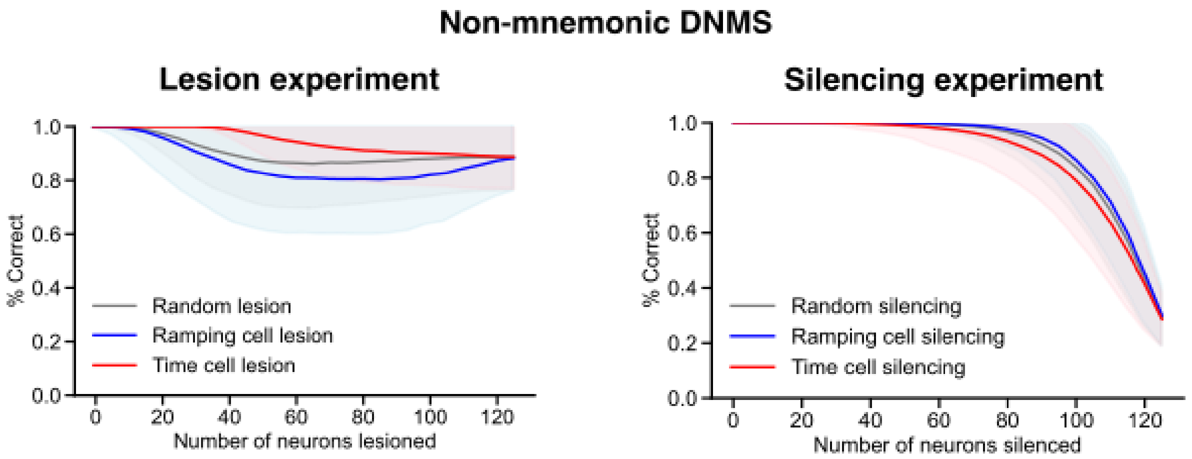

Figure S5: **Solving the non-mnemonic DNMS task does not rely on the recurrent dynamics during the delay period**. Same as Fig. 5 e-f), but for the non-mnemonic version of the DNMS task.
